# Hypnotism

**Published:** 1894-08

**Authors:** F. O. Hetrich


ARTICLE IV.

### HYPNOTISM.

BY DR. F. O. HETRICK.

Read before the Kansas State Dental Association.

The work of neurologists has in the past few years thrown new light upon the problem of hypnotism and given a foundation upon which to build theories. While I do not propose to discuss at length the psychological aspect of hypnosis, as it would be unprofitable in the time we have, I will refer to the fundamental principle as recognized by leading psycologists. I will quote from an article which I hope you have all read by Wm. Romaine Newbold Ph. D., of the University of Pennsylvania, the best short article I have seen upon this subject: "All mental states are accompanied and conditioned by physical processes in the cerebral cortex." Taking this for a fundamental principle, we have the problem where we have hope of its solution. This brings it into the realm of psycho-physiology. We will not go into further discussion of this principle, but let us take this as a basis for study the coming year and report at our next annual meeting. In the rest of this paper I shall, much of the time, use the form of question and answer. Many have asked me, What is hypnotism?

There are almost as many theories as there are theorists. This is a problem for solution, and, as I have changed my mind in regard to it in the last six months, I will not state as to what I believe it to be, but give you the theories of men who have studied it longer than I have been in the world. I may in the discussion hint at what I think of it. Our work to-day is more practical, and, just touching these we will pass on.

There are two general classes or schools, one of which believes that hypnosis is induced entirely by the action of the subject's mind, the other that it is largely the action of the operator's mind upon the subject. Mr. Braid, one of the first to study these phenomena systematically, says: "Hypnotism is induced by an impression made on the nervous centers by the physical and psychical condition of the patient, irrespective of any agency proceeding from or excited into action by another."

Stinson Jarvis, in an article in the *Arena* for December, 1893, infers almost the opposite. He cites a case wherein a patient was hypnotised without his knowledge, or at least yielding to hypnotic suggestion and swaying his body from side to side; stopped writing and other things without even knowing Mr. Jarvis, or that he was operating upon him. My experiments lead me to believe that they are both right and wrong. The mental and physical condition has much to do with the susceptibility of a patient to hypnosis, as also has the personality of the operator much to do with their yielding to that influence. If a patient comes to you with full confidence in you and in your ability to hypnotize him, your work is half accomplished before you begin. There are different ways of operating to bring your patient under hypnosis. The Donato method I do not like or use at all, as it is exhausting to patient and operator. The method or methods of Dr. Bernheim is the better in a large majority of cases, as it is a part of the suggestion you wish to use when the patient is fully under the influence; slower in its action, but so much kindlier and gentle. We owe much to this gentleman, as also we do to a number of others, among whom I might mention Messrs. Braid of England, Charcot of France, Chambard of France, Herbert Heidenheim of Germany, for a scientific study of the problem.

But perhaps I had better explain what the Donato method is. Professor Donato is a very wonderful man in many respects. He conceived the idea of instantaneous hypnosis and so formulated a method by which he placed the patients upon their knees before him; taking their hand slightly in his and telling them to press with all their might, getting every power of their body concentrated on that act and their thought concentrated on the muscular holding of his, he would suddenly spring back and throw a glance into their eye and commence to swing his body, and if the eye followed his movement, the patient would soon be under the influence. He has had marvelous success in that line.

I like the method suggested by Dr. Bernheim, as it wins your patient to believe that you will be gentle with him, and, as I said, is along the line you wish to suggest to the patient as you find him yielding to the influence. I shall demonstrate several different methods in the clinic room and not speak of them here.

Dr. Bernheim's means of concentration of the thought either through the eye, or counting the pulse, listening to the tick of a watch, or just simply closing the eye, then by a gentle, soft intonation of the voice working the patient to the point of yielding to that influence. Of course the monotony of it in many cases will produce, at least in a lower degree of hypnosis, the desired effect.

Many of you will remember my demonstration of the method of using the faradic current to obtund sensitive dentine at a clinic held by this society four years ago, in which we used a divided current of the negative pole. I thought that was going to be the solution for the obtunding of sensitive dentine. Some months after going home, I do not remember the exact time, I was working for a patient who had been very susceptible to the influence of this method of obtunding sensitive dentine. I had to fill the buccal and labial cavities, and, as you know, they are the most sensitive we ever find. That day it seemed to work better than usual, and, thinking I would see if I could make it absolutely painless, I said, "We will use just a little stronger current and see if we can't make this absolutely painless this morning." I did so, and in fact, as far as I could see at the time, I used the full strength of the battery. Wishing to know just the strength of the the current after the excavation of the cavity was completed, I tried it, and you can appreciate my astonishment when I found there was no current; the wire was broken. I wanted to be sure about it, so I gave her the electrodes again and said, "I wish to excavate just a little more at the cervical margin." I had tried the cavity before I began to use electricity to see if it was sensitive, and it was so sensitive I could scarcely wipe it out with cotton. So the electrodes were applied and she rested back in the chair and I excavated the cavity without a particle of pain. You can imagine how I felt; after having experimented with different batteries and different strengths of current, and there was the work of a year absolutely wasted. But a gentleman whose office is on the same floor with me, a physician and a bright fellow, quite a student, and who is in my office about one-third of the time, was informed of this phenomena. I said to him, "We have got to have another solution to our electrical problem; it is evidently hypnotic influence." He said, "I advise you to let hypnotism alone."

So I commenced pumping my professional friends among the physicians until I had pumped them all dry on hypnotism, and was advised almost invariably by them to let hypnotism alone, and there was a great mystery surrounding it, and it was not wise "to enter here." The re-result was that I kept at this little man until we began the study, as far as we could get hold of any books, and studied a number of authors on psychology. Then we began experimenting with hypnotism.

That is the way I first began to think anything of hypnotism. I had had, just as the large majority of men and women of this country, a sort of unholy idea of it. I thought it was not a thing that we ought to fool with or it would make a fool of me and all that I hypnotized.

We were surprised at the results we attained, while we did not proceed in a systemstic manner as we do now. My first experiments along this line were conducted in .company with this gentleman, and I wish to give him due credit; he is Dr. Herr, of Ottawa.

The first experiments we did together, he aiding at the chair at my side, and sometimes hypnotising the patient for me, About this time Dr. Phillebrown's first paper came out and I studied it, and I must confess I got a good deal of help from it along the line of Bernheim's method, and I wish to acknowledge that here, because it gave me a new line of thought. From that time to this I have been working along a little different line than is ordinary in hypnotism. That is, before that, I had tried to hypnotize every patient as far as I could take them. Get them as far under the hypnotic influence as I could possibly do. In studying the doctor's paper I realized for the first time the importance of the different degrees in hypnotism, and about that time I secured a chart of Messrs. Paul and Chambard on somnambulic life, treating hypnotism as artificial somnambulism. I received a great benefit along the line of study that I pursued for the last year and a half from that. While we can not draw positive lines and say that these are the only degrees of hypnotism, yet there are stages that are more readily recognized than others. But the thought came into my mind that every patient comes into your office prejudiced, at least ninty-nine out of every hundred. You take children from 5 to 8 years old, they come to your office, having heard some great story of how some other child has sat there and had a tooth ground down with one of those red-hot buzz-saw machines, and they come in trembling. They come dragging them up-stairs, and at times we see a conglomerated mass of head feet and legs come bursting into your office door. It is simply because they have associated the idea of dentistry and pain the operating chair and pain, the dental engine and horror.

Now, my work began along this line; taking the law of reintegration, which Sir William Hamilton recognized and gave a name. The law that "all ideas that have once been associated will always appear together." That is the substance of it.

I have gone along the line of disassociation of those ideas, using just enough hypnotic influence to disassociate the idea of the dental engine and pain. In many, many cases that is all that is necessary.

The highest faculty that the mind has recognized is consciousness, and there are many stages of hypnosis before consciousness is lost; they have never been classified, but along the line of work I have been doing, we do not take the patients to even where they lose their will. Why have I done this? you say. Simply because the general public have a prejudice against hypnosis. The great mass of the general public have read some newspaper article that emanated from the pen of some reporter that had no foundation whatever in fact. He was hard up for a subject and so he wrote upon some fad, and the fad happened to be hypnotism, and he concocted out of little or no fabric quite a story, and they have read that and taken it as gospel truth. They have never studied the question. They say: "What, put yourself in that man's power? Not much; not in anybody's power!" But if you can show them that they do not lose their will, that it is not necessary to take them to the extent that by hypnotic suggestion you can tell them at some other time to go and do certain acts, and they would go and do it in spite of themselves and not know why—to accomplish that, we have to take them to the degree at least of where consciousness is lost—you have removed one of the obstacles in your way as a professional man.

Now, hypnotism has been for years, until Drs. Gray and Bernheim and some of those others have taken up the question and given it scientific study, in the hands of irresponsible parties who went about the country giving lectures and performing these wonderful feats under the name sometimes of spiritualism, and sometimes hypnotism and sometimes mesmerism, until professional men have been afraid to touch it, fearing it would injure their standing. But I don't care anything about that, if I can relieve suffering.

Now, while I admit danger, while I know there is danger, from several sources, yet you will also admit there is danger from many things you use, and yet you use them for the greatest good to the greatest number. Take chloroform, for instance, nitrous oxide, and arsenic—we had a case of that here this afternoon. How many of you expect to give up arsenic entirely because some gentleman ill-advisedly used it in that way? I don't think any of you will.

And so, taking the priniciple of the disassociation of ideas, I have worked upon that plane, and it has been a marvel to me how much can be accomplished.

Many times when your patients come in you can see the pallor of fear upon their faces. Perhaps this patient does not know you. They have heard of you, or someone has sent them to you, and they sit down and you can see them shaking, and they grasp the chair and every muscle is rigid. But you begin to hypnotize them and gradually the muscles relax, they sigh, and begin to rest, and all through that dental operation, instead of pain, you suggest to them: "Now, just sit quietly here and have a nice rest while I am operating for you." "This will not hurt you." You have to keep repeating that suggestion and repeating it and repeating it. At first I did not, I thought it was not necessary, but I found they would come out of the analgesic stage, and so, to save time, I keep repeating it all the time, "This won't hurt you," "This won't hurt you." "Just sit here quietly," or repeat the word "sleep" or whatever word I use.

Now we come to the matter of suggestion. My professional brother, do you realize, do I realize, can we comprehend the fact, how far we by suggestion influence each other? It is almost like comprehending thought, or comprehending God, it is almost impossible, although we may in time. The question comes to each of us, how far outside of the hypnotic influence do we influence each other, or are we at any time free from hypnotic influence? I believe that every man and woman to a certain degree is an hypnotizer; others greater, others less.

I will not repeat the word "impossible," for I had a motto presented to me not long ago that has been of much benefit especially along the line of psycho-physiological research. It is a quotation from Arrage, wherein he says: "He who outside of pure mathematics speaks the word 'impossible' lacks prudence." I wish we might think of that in our discussion, and I hope some of you, if *you do not believe* in hypnotism, will be free to ask me questions and to oppose me with all your might, so far as that is concerned, that we may get at the truth. But let us not say that a thing is impossible because we can not do it.

I am not before you as a professional hypnotist, and in my clinic this afternoon I do not come here to give you a show, and I did not come here to perform on anybody who wants to play the "smart Aleck," because it is out of the question to hypnotize a person if they hold back and do not yield themselves fully to the influence.

Many people ask, "Can everybody be hypnotized?" I believe that everybody can be hypnotized by some one. I do not believe a man, woman or child lives that somebody cannot hypnotize under some conditions. Of course, that statement is broad and is disputed by many scientists. I am not a scientist. Perhaps, I should qualify the statement that every one can be hypnotized by saying, to a certain degree, some degree, to a degree of quieting and soothing, and I have never yet, where a person fully gave himself to my control, failed to in a marked degree reduce the sensibility of a sensitive tooth.

Now, I make these broad statements that they may provoke discussion, but I make them also because I believe them. I do not want to be understood as being a crank upon the subject, but I do want to be understood as being a champion of those methods which will lead us to be more careful of the feelings of our patients. We go along in this world in such a hurried manner, I shall call it. As Dr. Patterson and Dr. Bergstresser said this morning, we do concentrate our whole minds upon our business when filling a tooth, we get such a sixty-miles an-hour gait upon us. Let us remember there is something better than simply dollars in this world. We have too much of the commercial idea of dentistry.

You might right here ask me, Does hypnotism pay? I shall say, as a financial affair, it is a failure. It could be made to pay; if you wanted to start out and become an advertising quack, it could be made to pay beautifully. The reason it does not pay is because it takes so much time. But does it pay in other ways? Yes, a thousand times yes, because you relieve suffering and you take dread away.

What did Professor Blake say here last night? How it brought up to my mind many a child that had come to me and as he has come to your office. You can see that boy as he walked in there, dreading a dentist since he was a little child. And for that matter, how many of you gentlemen dare go now and sit down in a chair and have a tooth filled without having the shivers creep up your spine?

This is where hypnotism is to step in. Would you use it on all patients? By no means; I would not use it for a patient that did not need it. I have not time to do that. Would you use it for a patient that wanted to try it just as an experiment? By no means; I have not time to do that I am too busy. Would you, as you went out evenings to your friend's houses, allow them to make you the show of the evening? By no means. I have been invited to do that time and again and refused.

There is one danger of hypnotism that I wanted to speak of. That is, a person attempting to hypnotize that has not perfect control of himself. You do not know what patient may go beyond the seage that you think they are going to and go into the stage of lethargy. We had a scientific club at our town, and a number of the students or members met at my office and I read a paper before them on hypnotism and also gave a practical demonstration of the effect of one mind over another in the hypnotic state by blindfolding a man who was susceptible, and I had him read what was on a chart on the wall, clear away from him and clear away from them. They thought they would try it, so one evening I was hurriedly sent for to go over to a club where one of them boarded, and one fellow had hypnotized another and he got scared and was pretty near insane, because the boy went into a state of somnambulism and lethargy and they could not wake him up. After the influence of the hypnotist began to wear off, he became troubled and excited. Those of you who have not the most cool and corageous character had better let hypnotism alone. That danger, however, is not great, because there is not one in a great many who will go to that stage easily.

Who should hypnotize? Well, I will tell you of one man who should not hypnotize. That is, a man who is known to be of immoral tendencies and who has not perfect control of himself, and who might in the heat of passion commit a crime upon the person of a lady patient that he might and she might regret all their lives. He should be prohibited by the severest penalties.

Whom would you use it for most and in what cases? For children, first; begin with the children. How many times that problem has vexed the dental mind! How many times does it vex you? You have Mrs. General

Somebody in the chair. We don't work for anybody but generals' and judges' and senators' wives, daughters and sons. She is in the chair. We hear the yell coming up stairs; we know what is coming, what is going to be done. Here comes one of those incorrigible youngsters. Would you hypnotize him, or would you take advantage of the little fellow by your superior size and grasp him by the neck, lead him over to the chair, shove him down into it, have his mother hold his hands and someone else hold his feet, force the mouth open and jerk the tooth out, as has been done many times? Or would you prefer the kindlier but much slower plan? Mrs. General may have to wait a little while, while you just gain the confidence of that child enough to get him to stay in your office. The mother comes in and slams the door and puts her back against it and she knows she has got the boy. What are you going to do in that case? We have all had them. I will tell you what I do, and what hpynosis has done for many of those cases, or hypnotic suggestion. I go in and talk to the boy or girl a few moments and gain its confidence; don't say anything about dentistry, talk about something else. And if it is a child I know, I talk about some subject in common that we know about, and the little fellow begins to lose that wild look, and you tell him to be seated a little bit, and we will see what needs to be done. After you get through with Mrs. General, explain to her you may have to cut her sitting a little short. You may have been nearly through, and she is one of those nervous patients that don't like to wait with the rubber dam in her mouth. She wants her work done right then. It may cause you a little annoyance; let her wait, while you are waiting on the other.

Dr. Shulze: Couldn't you hypnotize Mrs. General while you are waiting on the other.

Dr. Hetrick: Perhaps she is hypnotized; if she is, she is as quiet as a lamb. I am giving you an experience, though, where I am supposing she is not hypnotized. She could be hypnotized, but in case she is not, let her wait a little. What would you do with the boy? He has been sitting there until he is over this awful feeling that something were going to happen. You get to talking to him and you take a little mouth-glass, and you walk up to him and you tell him to look into it. I am giving this not in the form of a paper, but more as a practical example of how I do that thing. I hold it before his eyes and say, "You look in there, and in a little bit you will see a sheep jump over the fence there," and he begins to look, and you keep saying, "Now watch for the sheep," and after a while he will see a sheep jump over the fence. You see his eyes lifting, you are careful to keep the glass a little above his eyes, and he will watch those sheep, and when he sees about a hundred and fifty of them go, he gets sleepy, and then you say, "I am going to put medicine in that tooth, and it won't hurt you," and you keep on in that way until you see the eyes get heavy.

Dr. Phillebrown says not to use passes, they are not necessary. I do not always use the same method, but with children I always use passes. Of course, in teaching a Sunday-school class, or talking to a boys' brigade, or anything of that kind, if you keep your hands and feet going, they watch your motions. So let us bring our knowledge of children to bear on the handling of them in the dental office. You pass your hands in front of him like this, and say, "Your eyes are getting so you can hardly open them; they are just about as heavy as lead," and he will shut his eyes, and you say, "You can't open them," and nine times out of ten he can not, and your child is under your control. The operation is performed without pain. That is an extreme case and requires taking to a degree of hypnotism that I do not ever have to take that child again unless I extract teeth for him. As soon as I gain his confidence, I have a lifelong friend, and one who has confidence in me. Has it paid? Operations from that on are comparatively easy, the child has had the dread of the dental room taken away from him. Is it worth trying for, gentlemen? I am not here for you to take simply my word. As I

say, I expect to demonstrate this to you this afternoon. I do not do it because I want to pose as a hypnotist; I do it for the purpose that some of you, who have not investigated this subject may learn how, in a kindly way, to relieve a great deal of suffering for your patients.

I will mention one or two cases, although I do not like this thing of stating cases, because, in hypnotism, at least, it smacks a little bit of braggadocio; but I will use them in illustration of my subject for what they are worth.

There were three very marked cases. One of a lady; I was called about half past nine in the evening, and I found her walking the floor and the sweat standing out upon her brow. She was nearly wild with pain, and it made me nervous to look at her. Her face was swollen until her eye was nearly shut, with an ulcerated tooth; just in that stage when pus-formation is taking place and the pain is excruciating. I said to the lady, "There is not anything I can do to give you as much relef as to hypnotize you." "Why, I never heard of such a thing. Oh, well, my father use to hypnotize people. Do you think you can stop my pain?" Well, I will not go into details, I will simply say, with the patient in that condition, I hypnotized her until she lost consciousness, and suggested to her that she go to bed and sleep all night, excepting that she would wake up three times to see that her baby was all right. Then I suggested to her that she would immediately go into her bed-room and retire. She did so, and as she went out of the room her husband said, "I never saw her mind anybody that quickly before." This was about a year ago. To be sure that my patient would have rest while she was in her couch, I went in and hypnotized her to the degree where she lost consciousness, and told her she would wake up about five or six o'clock the next morning. I went to see her the next morning. She had slept the whole night except the three times I told her she would wake up to see the baby. And I hypnotized her again and opened the abscess, which was ready to let the pus out. She had not suffered a particle of pain the whole night. That may sound a little bit "snaky" to some of you, but that is the fact.

This is a case evidently of psycho-therapeutics. I think there is no doubt but what the mind had power over the body in that case.

The lady was just recovering from an attack of bilious fever and was not able to come to the office for nearly a week. After I opened the abscess and let the pus out, it healed very rapidly and the tooth has been saved since that time. However, I have never with that patient had to hypnotize her since the time I opened the abscess, as all I need to do to fill a sensitive tooth for her is to just tell her to shut her eyes and repeat any one word, I don't care what it is, and it won't hurt her. I should have stated, the oftener a patient is hypnotized, the more susceptible they become to the influence.

Case two was that of a lady who had been a source of a great deal of trouble to me because of very sensitive teeth, with a low state of health and a case of hysteria that made it very hard to do dental operations for her. I had to keep her teeth patched up with cement and just wiped them out many times, but could not prepare the cavity. She had been troubled with insomnia to a fearful extent and she had traveled over many parts of the country especially to get relief for insomnia. In this case I hypnotized her, not until she lost consciousness at any time, and her teeth, some are crowned and others filled with gold where a crown was not required—filled with permanent filling throughout, and until that time I was never able to put a permanent filling in her mouth. She suffered no pain. And as I have been studying along the line of psycho therapeutics, I thought I would try a little suggestion for her insomnia. One day, near the close of the operation—and, by the way, she would sit down and rest during the entire operation and when she got through with it I would keep suggesting to her, "You will feel better when you get through with this than you do now." "You will feel rested," so I had kept her under the influence all the time in the sitting. One operation consumed four hours. I do not usually keep a patient over an hour and a half or two hours, but there was an operation I could not finish in less time than that, and at the close of that operation the thought came into my mind to try hypnotic suggestion for her insomnia.

That was in the early part of her coming to the office to have her permanent work done, and before she had ceased coming, by suggestion I had her sleeping all night long.

Now, I don't need to cite cases of this kind to you; the books are full of them. Of course, many times they are disputed and we may have to change our ideas of these matters many times. But, as Mr. Newbold, in his article, says, "It is out of the ruins of many theories that truth rises."

The other case is that of a patient with whom I came the nearest to losing my nerve, as they say, than any I ever had. It was one of those cases where there is danger of losing perfect self-control. It was a patient whom, were I to mention her name, there are four or five dentists in this room would recognize, she is very nervous. She was opposed to hypnotism. I could not wipe the cavities out with cotton without some pain, and excavating would nearly set her frantic. They were both of them baccal cavities. This was done last Friday and this is one of the most marked cases I ever had to treat. I had treated the cavities with nitrate of silver for several weeks, she having objected all the time to being hypnotized. Finally I said to her: "There is no use fooling along in this way, because you will either have to wear this black patch on your teeth (as she called the place where the nitrate of silver acted), or else let me hypnotize you and fill it." She said: "I am going visiting and I am not going with that on," so she consented to be hypnotized and I did so. I did not use the glass at all. Just simply told her to close her eyes and repeat the word "sleep," and I said: "Now your feet are beginning to feel heavy and asleep, and it is gradually moving up through your knees, and it is pretty nearly up to your body, it is getting close to your hands, and just kept repeating the word 'sleep,' and it don,t matter what I say to you." I kept up a monotonous talk in about this tone of voice until finally the first thing I knew her hands fell just like they were lead and her feet dropped, for she had lost all power of controling herself and I had a case of lethargy. I filled those teeth and did so hurriedly—that is, not to the extent of slighting the work, but as rapidly as I could—and suggested to her of course she could hear everything I said; I said to her: "Now, just sit here and sleep until I am through and this will not hurt you a particle." I had to put the clamp upon the gum, probably for the thirty-secondth of an inch, and it did not hurt her a particle. I filled the teeth rapidly, without much thought of her, and when I came to wake her up she regained consciousness comparatively easily. She said: "You do not mean to tell me that those teeth are filled? I thought you were going to fill my teeth." I handed her the glass and showed her the two fillings.

Those are the three marked cases I have had. We will all have failures. There is no cure-all in this world. We will all have them in any line of our work, but they should only be spurs to us to make us more determined that, as far as in us lies, we will always do the best possible for our patients. If any of you have any questions, I will try to answer them.—*Western Dental Journal.*

---

ARTICLE V.

